# IFN-γ Upregulates *Survivin* and *Ifi202* Expression to Induce Survival and Proliferation of Tumor-Specific T Cells

**DOI:** 10.1371/journal.pone.0014076

**Published:** 2010-11-22

**Authors:** Mary Zimmerman, Dafeng Yang, Xiaolin Hu, Feiyan Liu, Nagendra Singh, Darren Browning, Vadivel Ganapathy, Phillip Chandler, Divaker Choubey, Scott I. Abrams, Kebin Liu

**Affiliations:** 1 Department of Biochemistry and Molecular Biology, Medical College of Georgia, Georgia Health Sciences University, Augusta, Georgia, United States of America; 2 Immunotherapy Center, Medical College of Georgia, Georgia Health Sciences University, Augusta, Georgia, United States of America; 3 Department of Environmental Health, University of Cincinnati, Cincinnati, Ohio, United States of America; 4 Department of Immunology, Roswell Park Cancer Institute, Buffalo, New York, United States of America; Universidade de São Paulo, Brazil

## Abstract

**Background:**

A common procedure in human cytotoxic T lymphocyte (CTL) adoptive transfer immunotherapy is to expand tumor-specific CTLs *ex vivo* using CD3 mAb prior to transfer. One of the major obstacles of CTL adoptive immunotherapy is a lack of CTL persistence in the tumor-bearing host after transfer. The aim of this study is to elucidate the molecular mechanisms underlying the effects of stimulation conditions on proliferation and survival of tumor-specific CTLs.

**Methodology/Principal Findings:**

Tumor-specific CTLs were stimulated with either CD3 mAb or cognate Ag and analyzed for their proliferation and survival *ex vivo* and persistence in tumor-bearing mice. Although both Ag and CD3 mAb effectively induced the cytotoxic effecter molecules of the CTLs, we observed that Ag stimulation is essential for sustained CTL proliferation and survival. Further analysis revealed that Ag stimulation leads to greater proliferation rates and less apoptosis than CD3 mAb stimulation. Re-stimulation of the CD3 mAb-stimulated CTLs with Ag resulted in restored CTL proliferative potential, suggesting that CD3 mAb-induced loss of proliferative potential is reversible. Using DNA microarray technology, we identified that *survivin* and *ifi202,* two genes with known functions in T cell apoptosis and proliferation, are differentially induced between Ag- and CD3 mAb-stimulated CTLs. Analysis of the IFN-γ signaling pathway activation revealed that Ag stimulation resulted in rapid phosphorylation of STAT1 (pSTAT1), whereas CD3 mAb stimulation failed to activate STAT1. Chromatin immunoprecipitation revealed that pSTAT1 is associated with the promoters of both *survivin* and *ifi202* in T cells and electrophoresis mobility shift assay indicated that pSTAT1 directly binds to the gamma activation sequence element in the *survivin* and *ifi202* promoters. Finally, silencing *ifi202* expression significantly decreased T cell proliferation.

**Conclusions/Significance:**

Our findings delineate a new role of the IFN-γ signaling pathway in regulating T cell proliferation and apoptosis through upregulating *survivin* and *ifi202* expression.

## Introduction

Data from extensive studies of human cancer patients and animal models in the last two decades strongly support the existence of an intrinsic cancer immunosurveillance system that in the absence of external manipulation functions to protect the host against tumor development [1], [2]. In human cancer patients, a high level of tumor-infiltrating lymphocytes and immunological effecter molecules in the tumor microenvironment is often correlated with prolonged survival, decreased disease recurrence and delayed metastasis [3], [4], [5]. Consistent with these observations, cytotoxic T lymphocyte (CTL) immunotherapy has been shown to effectively suppress tumor development in certain cancer patients [6], [7], [8], [9], [10], [11], [12]. Adoptive CTL transfer immunotherapy provides the opportunity to overcome intrinsic immune suppression and tolerance mechanisms by enabling the *ex vivo* selection and expansion of highly tumor-reactive CTLs and has emerged as one of the potentially effective treatments for patients with metastatic cancer. However, a major obstacle to the development of effective CTL adoptive transfer immunotherapy for patients with cancer and other diseases has been the lack of persistence of the transferred CTLs in the host [13], [14], [15], [16]. Although telomere lengthening and telomerase activity have been linked to CTL function and persistence [17], [18], [19], the molecular mechanisms underlying the lack of persistence of the tumor-reactive CTLs in the tumor microenvironment is largely unknown. Studies in human cancer patients with a nonmyeloblative but lymphodepleting chemotherapy regimen before CTL adoptive transfer failed to improve CTL persistence following transfer [14], suggesting that factors that were intrinsic to the CTLs, perhaps the conditioning of CTLs prior to adoptive transfer, might be responsible for the lack of CTL persistence *in vivo*.

One of the procedures of CTL adoptive transfer immunotherapy is *ex vivo* expansion of the tumor-specific CTLs with CD3 mAb prior to adoptive transfer [6], [13]. Because adequate stimulation mediates T cell function and survival [20] and polyclonal stimulation through the TCR/CD3 complex has the potential to induce T cell anergy and apoptosis [21], [22], we hypothesized that expanding the tumor-specific CTLs with CD3 mAb prior to adoptive transfer, although effective for T cell expansion, might not optimally condition the CTLs to survive *in vivo* after transfer. To test this hypothesis, we used experimental metastasis and CTL adoptive transfer mouse models to elucidate the molecular mechanisms underlying tumor-specific CTL persistence in the tumor microenvironment. Our results suggest that the IFN-γ-signaling pathway may play a critical role in mediating CTL persistence in an autocrine manner by directly regulating *survivin* and *ifi202* expression during T cell activation.

## Methods

### Mice

Female BALB/c (H-2^d^) mice were used in all studies and were purchased from the National Cancer Institute (Frederick, MD). Mice were housed in the Medical College of Georgia animal facility. Experiments and care/welfare were in agreement with federal regulations and an approved protocol (Protocol # 05-12-728*B) by the MCG/IACUC committee.

### Cell lines

The cell line CMS4, kindly provided by Dr. A. Deleo (University of Pittsburgh, Pittsburg, PA), is a primary soft tissue sarcoma of BALB/c (H-2^d^) origin. CMS4-met is a highly metastatic subline derived from CMS4 tumor cell line as described [23]. 5KC is a T cell hybridoma [24] kindly provided by Dr. P. Kraj (Medical College of Georgia, Augusta, GA). P815 was obtained from ATCC and used as a negative control cell line for CTL specificity.

### Tumor-specific CD8^+^ CTLs

CD8^+^ CTLs reactive against the CMS4 tumor cells were established from wild-type (wt) BALB/c mice as previously described [25]. Briefly, mice were immunized with viable CMS4 tumor cells (5×10^5^ cells given s.c. on one flank) coadministered with anti-CTLA-4 mAb [affinity-purified hamster anti-mouse clone UC10-4F-11, hybridoma line provided by J. Bluestone (University of California, San Francisco, CA)] at 100 µg/inoculation/mouse given i.p. on days 0, 3, and 6 post-tumor transplant. Mice that were exposed to this regimen and failed to develop primary tumors were re-challenged on the contralateral flank with tumor cells in the absence of CTLA-4 mAb. Splenic CD8^+^ cell lines were derived from re-challenged mice, which showed little to no additional tumor growth. CTL cultures (2×10^5^ cells per well) were propagated *in vitro* in 24-well plates by stimulation with irradiated (200 Gy) CMS4-met tumor cells (1×10^5^ cells per well) as a source of cognate Ag, irradiated (20 Gy) syngeneic BALB/c splenocytes (5×10^6^ cells per well) as feeder cells and IL-2 (60 IU/ml. Tecin, Hoffman-LaRoche, Nutley, NJ). CTLs were purified from dead tumor cells and spleen cells at the end of the stimulation cycle over a Ficoll gradient and re-stimulated weekly with freshly prepared irradiated tumor cells and irradiated spleen cells. For CD3 mAb stimulation, 24-well plates were coated with anti-CD3 and anti-CD28 mAbs (BD biosciences) at 1 µg/well in 250 µl PBS overnight at 4°C. CTLs were then seeded in the mAb-coated plates as in the tumor-stimulated CTLs. IL-2 (60 IU/ml) was also added to the culture.

### In vitro cytotoxicity assays

CTL cytotoxicity was assessed by ^51^Cr-release assays as previously described [26]. Briefly, target cells were labeled with Na_2_^51^CrO_4_ (ICN Biomedical). CTLs were recovered from culture by centrifugation over a Ficoll-Hypaque gradient. CTLs and radiolabeled target cells were then coincubated in 96-well, U-bottom plates at various effector/target ratios. After incubation for 18 hr, supernatants were collected using a Supernatant Collection System (Skatron Co., Sterling, VA). Radioactivity was quantitated using a γ-counter. Percent specific ^51^Cr-release was calculated according to the following formula: % specific lysis  =  [(experimental cpm-spontaneous cpm)/(total cpm-spontaneous cpm)] x 100%. Total ^51^Cr-release was obtained by adding 0.2% Triton X-100 (final concentration) to the wells. Data are reported as the mean ± SD of triplicate wells, and representative of one of two separate experiments.

### Experimental lung metastasis model

Female BALB/c (H-2^d^) mice were obtained from the National Cancer Institute (Frederick, MD). CTL adoptive transfer immunotherapy was carried out essentially as previously described [27]. Briefly, CMS4-met tumor cells (2.5×10^5^ cells/mouse) were injected into mice i.v. in 100 µl HBSS. CTLs were stimulated for 4 days and purified from the culture mixture as described above and injected to the tumor-bearing mice three days later. Mice were sacrificed at the time points indicated. Lungs were inflated with a 15% solution of India ink, resected, and fixed in Fekete's solution (60% ethanol, 10% formaldehyde, and 5% glacial acetic acid). The number of lung nodules was enumerated in all four lobes in a single-blinded fashion. Values exceeding 250 nodules were considered too numerous to count accurately and, therefore, were reported as ≥250.

### Histological Analysis

Lungs were excised from tumor-bearing mice and fixed in 10% formalin. Sections were cut from formalin-fixed, paraffin-embedded, tumor-bearing lungs, and stained with hematoxylin and eosin.

### Persistence of adoptively-transferred CTLs *in vivo*

CMS4-met tumor cells (2×10^5^/mouse) were injected i.v. into naive mice. Five days later, CTLs were labeled with CFSE using the CellTrace^™^ CFSE Proliferation Kit (Invitrogen) according to the manufacturer's instructions. CFSE-labeled CTLs (1×10^6^/mouse) were injected i.v. into tumor-bearing mice. Four days later, lungs were removed and digested with an enzyme mixture containing hyaluronidase (0.1 mg/ml), collagenase (1 mg/ml), and DNase I (30 U/ml), all obtained from Sigma-Aldrich, to generate single cell suspensions. The cell suspensions were then analyzed by flow cytometry to detect CFSE-positive cells.

### Proliferation and apoptosis assay

CTLs were labeled with CFSE as described above. CFSE-labeled CTLs were then stimulated with either the H-2L^d^-resticted peptide AH1 (SPSYVYHQF) [23] as antigen and irradiated spleen cells as feeder cells or with the CD3 mAb plus CD28 mAb and recombinant IL-2 as described above. Cells were stained with Annexin V-Alex Fluor 647 (Biolegend, San Diego, CA) and PI three days after stimulation and analyzed by flow cytometry. Cell proliferation was then analyzed with Modfit program (Verity Software House, Topsham, ME) to compare the division times of the cells under both stimulation conditions.

### ^3^H thymidine incorporation assay

CTLs were mixed with irradiated CMS4-met cells and splenocytes as described above and seeded in flat-bottom 96-well plates at a density of 5×10^4^ CTLs/well in 100 µl medium. Mixtures of irradiated tumor cells and splenocytes without CTLs were used as the background control. For CD3 stimulation, CTLs at the same density were seeded in CD3 and CD28 mAb-coated 96-well plates. Approximately 24 hrs later, ^3^H-thymidine (10 µCi/well) was added to the cell cultures and incubated for another 24 hrs. Cells were harvested and counted for ^3^H thymidine incorporation ^3^H thymidine incorporation (CPM counts) was calculated by the formula: CPM counts of the culture mixture of irradiated tumor cells, feeder cells and CTLs mixture – CPM counts of the culture mixture of irradiated tumor cell and feeder cells. Cell growth rate was also measured by MTT assay (ATCC, Manassas, VA). Cells were seeded in 96-well plates in 100 ml culture medium for approximately 48 h. MTT (10 ml) were then added and cultured for another 2–3 h. Cells were lysed with detergent and measured at OD570 according to the manufacturer's instruction.

### Gene Silencing

Scramble shRNA (Cat# sc-108080) and ifi202-specific shRNA-expressing lentiviral particles were obtained from Santa Cruz Biotech (Cat# sc-40698-V). 5KC cells were transduced with the viral particles and selected for stable lines with puromycin according to the manufacturer's instructions.

### RT-PCR Analysis

Total RNA isolation and cDNA synthesis was carried out as previously described [28]. For isolation of total RNA from stimulated CTLs, tumor-stimulated CTLs were purified with CD8 mAb-conjugated magnet beads (Invitrogen) and lysed for total RNA. CD3 mAb-stimulated cells were harvested from the plates and lysed for total RNA. The cDNA was then used as templates for PCR amplification of various genes using gene-specific primers. The following parameters were used: 30 sec at 94°C, 30 sec at 60°C, and 1 min at 72°C. PCR amplification for all genes was first performed using 30 cycles. The PCR reactions were then repeated using less or more cycles for optimal amplification. PCR primers are listed in table 1.

**Table 1:** PCR primer and EMSA probe sequences.

Name	Sequence	PCRCycle #
Perforin	F: 5′-CCACAGGCTCATCTCCTCCTATG-3′R: 5′-TCCACCAGACCAGGGTTGC-3′	30
Granzyme A	F: 5′- CCTGAAGGAGGCTGTGAAAGAATC -3′R: 5′- CCTGCTACTCGGCATCTGGTTC -3′	30
Granzyme B	F: 5′-GCCCACAACATCAAAGAACAGG-3′R: 5′-CCAGAATCCCCCCGAAAGG-3′	30
FasL	F: 5′-CTTGGGCTCCTCCAGGGTCAGT-3′R: 5′-TCTCCTCCATTAGCACCAGATCC-3′	30
IFN-γ	F: 5′- ATGGCTGTTTCTGGCTGTTACTG -3′R: 5′- GCTTCCTGAGGCTGGATTCC -3′	30
TNFα	F: 5′- TGACAAGCCTGTAGCCCACG -3′R: 5′- GACTCCAAAGTAGACCTGCCCG –3′	30
LTα	F: 5′-TGCCAGGACAGCCCATCCAC-3′R: 5′-TGAGCAGGAACACAGCCCC-3′	30
LTβ	F: 5′-TGGATGACAGCAAACCGTCG-3′R: 5′-AACGCTTCTTCTTGGCTCGC-3′	30
LIGHT	F: 5′-GGCTGGAACAGAACCACCG-3′R: 5′ -CCAAGTCGTGTCTCCCATAACAGAG-3′	30
ifi202	F: 5′- CTCCAACTCTTCTCCTTTCACCTG -3′R: 5′- TGGCTCTTCACCTCAGACACG -3′	28
ifi202 (ChIP)	F: 5′- ATGAGAAGGAGGTGGGGATGAGTG -3′R: 5′- CCAGAAAAATAGTCACAAGGACGC -3′	35
HPRT	F: 5′- CTTGCTGGTGAAAAGGACCTCTC -3′R: 5′- CAAATCAAAAGTCTGGGGACGC -3′	30
β-actin	F: 5′-ATTGTTACCAACTGGGACGACATG-3′R: 5′-CTTCATGAGGTAGTCTGTCAGGTC-3′	30
survivin	F: 5′-GCAAAGGAGACCAACAACAAGC-3′R: 5′-CCACAAAACCAAAGAGAGGTAGCC-3′	30
survivin (ChIP)	F: 5′- AGCAAGCAGTGGGAGCACGG -3R: 5′- CCAACTTCAGGAGAGCGTTTATTG -3	38
Survivin (EMSA)	F: 5′-TAAAGACTTCCCAGAATTCCAA-3′R: 5′-TTGGAATTCTGGGAAGTCTTTA-3′	38

F: forward. R: reverse.

### Western Blot Analysis

Cells were lysed in lysis buffer (20 mM Tris-HCl, pH 7.4, 150 mM NaCl, 1 mM EDTA, 1% Triton X-100) plus proteinase and phosphotase inhibitor cocktails (Calbiochem, San Diego, CA) for 60 min on ice. Proteins were separated on a 4–20% SDS-polyacrylamide gradient gel, and transferred to PVDF membrane (Millipore, Bedford, MA). Anti-survivin (Santa Cruz Biotech), anti-pSAT1 (BD biosciences), anti-STAT1 (BD biosciences), and anti-β-actin mAb (Sigma) were used as the primary Abs, followed by incubation with horseradish peroxidase-conjugated anti-goat (Santa Cruz Biotech, Santa Cruz, CA), anti-mouse or rabbit (Amersham-Pharmacia, Sunnyvale, CA) IgGs. Antiserum to p202 protein, which detects both the p202a and p202b proteins has been described [29]. Immunodetection was performed using the ECL Plus kit (Amersham-Pharmacia).

### Cell surface marker analysis

Cells were incubated with biotin-conjugated anti-mouse IFN-γR mAb (BD biosciences), followed by incubation with PE/Cy5-conjugated Streptavidin (BD Biosciences, CA). Control preparations were incubated with the appropriate fluorescently labeled isotype-matched IgG. The stained cells were washed and analyzed by flow cytometry.

### Genome-wide gene expression analysis

Total RNA was isolated from cells using Trizol (invitrogen) and used for cDNA probe preparation. cDNA probes were synthesized using the FairPlay microarray labeling kit (Stratagene, La Jolla, CA). The cDNA probes were then labeled with Cy3 or Cy5 monofunctional reactive dye (Amersham Biosciences, Piscataway, NJ). The appropriate Cy3 and Cy5 labeled probes were combined and used to probe the processed mouse oligo microarray chips (approximately 36,000 sequences of transcripts). Fluorescent images were captured using a Genepix 4000 (Axon Instruments, Union City, CA). Both image and signal intensity data were loaded into a database supported by the Center for Information Technology (CIT) of NIH. Cy3:Cy5 intensity ratios from each gene were calculated and subsequently normalized to ratios of overall signal intensity from the corresponding channel in each hybridization. The normalized data were then extracted from the database as text files, and analyzed using computer software JMP (SAS Institute, Cary, NC). All microarray data are MIAME compliant and are deposited in GEO database (accession # GSE22890).

### Chromatin immunoprecipitation (ChIP) assay

ChIP assays were carried out essentially according to protocols from Upstate (Lake Placid, NY) as previously described [30]. Briefly, cells (approximately 3×10^6^ per assay) were fixed in 10% formaldehyde for 10 min at 37°C, lysed in SDS lysis buffer on ice for 10 min, and sonicated with a 60 Sonic Dismembrator (Fisher Scientific, Pittsburgh, PA). The lysate was pre-cleared with a mixture of 0.04% sheared salmon sperm DNA, 0.1% BSA, and agarose-protein A beads (Upstate) for 60 min at 4°C. The pre-cleared supernatants were then incubated with anti-pSTAT1 antibody (Santa Cruz Biotech) or mouse IgG mAb (5 µg antibody per assay) overnight at 4°C. A mixture (approximately 25 µl per assay) of shared DNA, BSA, and agarose-protein A beads was then added to the lysate and incubated at 4°C for 60 min. The beads were then extensively washed. The protein-DNA complexes were eluted from beads and the crosslinking was reversed. The DNA was purified from the eluted solution and used for PCR.

### Protein-DNA Interaction Assay

Electrophoresis mobility shift assays (EMSA) were carried out as previously described [30]. The end-labeled probes were incubated with nuclear extracts (20 µg) in the binding buffer [10 mM Tris-HCl, pH 7.5, 1 mM MgCl_2_, 0.5 mM EDTA, 0.5 mM DTT, 50 mM NaCl, 4% Glycerol, and 0.05 mg/ml poly(dI-dC).poly(dI-dC)] for 20 min at room temperature. For specificity controls, unlabeled probe was added to the reaction at a 1∶100 molar excess. Anti-pSTAT1 mAb (Santa Cruz Biotech) was also included to identify pSTAT1-specific DNA binding. Anti-pSTAT1 was incubated with the nuclear extracts for 30 min on ice before addition of labeled probes. DNA-protein complexes were separated by electrophoresis in 6% polyacrylamide gels in 45 mM Tris borate, 1 mM EDTA, pH 8.3. The gels were dried and exposed to a phosphoimage screen (Molecular Dynamics) and the images were acquired using a Strom 860 imager (Molecular Dynamics). The EMSA probe sequences are listed in Table 1.

### Statistical analysis

Where indicated, data were represented as the mean ± SD. Statistical analysis was carried out using two-sided *t* test, with *p*-values<0.05 considered statistically significant.

## Results

### Cognate Ag and CD3 mAb stimulation leads to different tumor rejection efficacy

**Figure 1:**
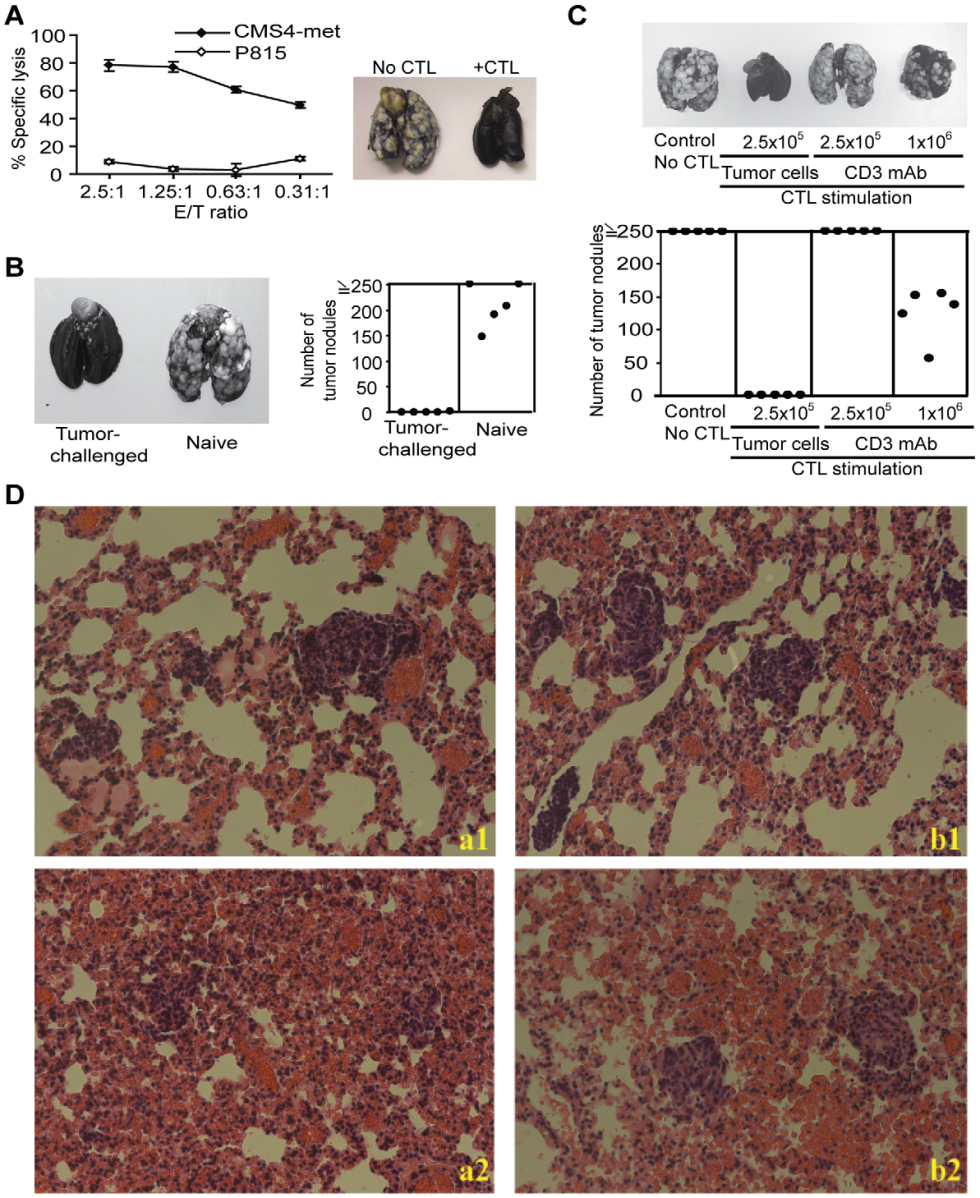
Antigen-activated CTLs exhibit greater tumor rejection efficacy than CD3 mAb-stimulated CTLs. **A**. Lytic sensitivity of CMS4-met tumor cells to tumor-specific CTLs. Left panel: *in vitro* CTL cytotoxicity assay. Lytic sensitivity was assessed by ^51^Cr release. Tumor cell line P815 was used as a negative target control for CTL specificity. Right panel: photomicrographs of representative individual lungs of mice without (No CTL) and with (+CTL) CTL adoptive immunotherapy. **B**. Anti-tumor immunological memory. Mice that received tumor cell transplantation and CTL adoptive transfer immunotherapy (tumor-challenged) as shown in A and naive mice were challenged approximately 90 days after CTL immunotherapy with the CMS4-met tumor cells and analyzed for lung metastasis. The number of tumor nodules were quantified and shown at the right. Each dot represents total tumor nodule counts from a single mouse. **C**. Tumor-activated CTLs exhibited much greater tumor rejection efficacy than CD3 mAb-stimulated CTLs. CMS4-met tumor cells were injected i.v. into mice. Three days later, saline (Control, no CTL) or tumor-specific CTLs were injected into the tumor-bearing mice i.v. at the doses indicated. CTLs were either stimulated with irradiated tumor cells or with CD3 mAbs and were adoptively transferred to the tumor-bearing mice. Shown are photomicrographs of individual lungs of mice (top panel). Bottom panel: quantification of total pulmonary nodules of individual mice as shown in top panel. Each dot represents the total tumor nodule counts from a single mouse. **D**. Histological analysis of lung metastasis. CMS4-met cells were injected into naïve mice iv, three days later, tumor cell-stimulated (a1 and a2) and CD3 mAb-stimulated (b1 and b2) CTLs were injected into the tumor-bearing mice, respectively. Lungs were excised one (a1 and b1) and three (a2 and b2) days after CTL adoptive transfer and subject to histological analysis with H&E staining.

Conventional CTL adoptive transfer cancer immunotherapy requires *ex vivo* expansion of tumor-specific CTLs with CD3 mAb before the CTLs are transferred to the patients [14], [31], [32]. However, the adoptively transferred CTLs often failed to persist in the patients [14], [33]. Although CD8^+^ T cells can be re-stimulated to rapidly expand using CD3 mAb, Ag-specific T cells often gradually stop responding to re-stimulation and enter a “senescence-like” state [34]. Therefore, we hypothesized that the lack of persistence of adoptively transferred CTLs in the host might be due, at least in part, to the stimulation conditions of the CTLs before adoptive transfer. To test this hypothesis, we used a well-established CTL adoptive transfer and experimental lung metastasis mouse model [23], [25], [35] to examine the effects of stimulation conditions on CTL persistence. These CTLs were specific for CMS4-met sarcoma tumor cells in a dose-dependent manner *in vitro* and exhibited potent tumor rejection efficacy against transplanted CMS4-met tumors *in vivo* (Fig. 1A). Furthermore, mice receiving CTL adoptive transfer immunotherapy also exhibited potent immunity against tumor re-challenge (Fig. 1B), suggesting the development of immunological memory in the tumor-challenged mice. Next, tumor-specific CTLs were stimulated with CD3 mAb in the presence of a co-stimulation signal (CD28 mAb) or with the cognate Ag (tumor cells) and analyzed for tumor rejection efficacy *in vivo*. As shown in Fig. 1C, the tumor-stimulated CTLs effectively rejected the transplanted tumor, whereas the CD3 mAb-stimulated CTLs exhibited poor anti-tumor efficacy even at a higher dose (Fig. 1C). Histological analysis of the tumor-bearing mouse lungs revealed that metastatic foci in the lungs are effectively destroyed by tumor-stimulated CTLs (Fig. 1D, a1&2), but not by CD3 mAb-expanded CTLs (Fig. 1D, b1&2). Taken together, our data suggest that *ex vivo* activation conditions at least partially determine tumor rejection efficacy *in vivo*.

### Both cognate Ag and CD3 mAb can effectively activate CTLs

**Figure 2:**
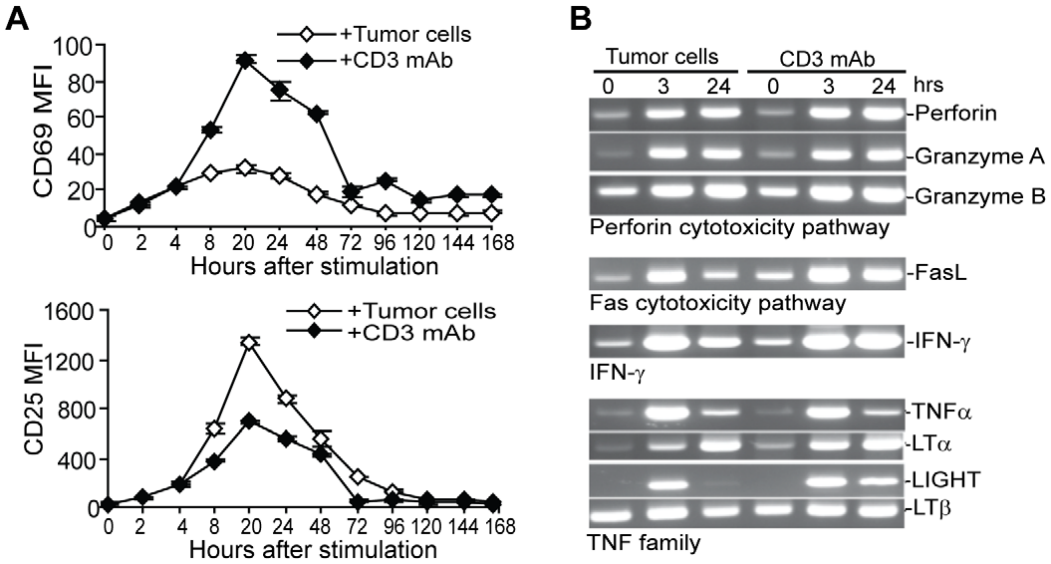
Both antigen and CD3 mAb effectively activate tumor-specific CTLs. **A**. Activation markers CD69 and CD25 expression kinetics. CTLs were stimulated with either tumor cells or CD3 mAb for the indicated time points. Cells were stained with CD8-specific mAb plus CD69- or CD25-specific mAbs, respectively. The CD8-positive cells were gated and measured for CD69 and CD25 mean fluorescent intensity (MFI). **B**. Analysis of expression levels of effector molecules of the cytotoxic pathways. CTLs were stimulated with either irradiated tumor cells or CD3 mAb and harvested at the indicated time points. RT-PCR analysis was carried out using gene-specific PCR primers as indicated. LTβ is also used as a normalization control.

Suboptimal stimulation of T cells can lead to anergy or senescence *in vivo* [22], [36], [37]. To determine whether the poor efficacy of CD3 mAb-expanded CTLs to reject the established tumor is due to induction of T cell anergy or senescence, CTLs were stained for cell surface expression levels of CD69 and CD25, two markers of T cell activation, at various time points after stimulation. Tumor cell-stimulated CD8^+^ CTLs exhibited a greater degree of CD25 activation but a lesser degree of CD69 activation compared to CD3-stimulated CTLs (Fig. 2A). However, the overall CD69 and CD25 activation kinetics are qualitatively similar between the Ag- or CD3 mAb-stimulated CTLs. Thus either tumor cells or CD3 mAb effectively activated the CTLs.

To determine whether the poor tumor rejection efficacy of the CD3 mAb-stimulated CTLs is due to lack of activation of cytotoxic effecter mechanisms, we compared activation of the cytotoxic pathways in the tumor-specific CTLs. The expression kinetics of key effecter molecules (perforin, granzyme A and granzyme B) of the perforin pathway, the Fas/FasL (FasL) cytotoxic effecter pathways, the IFN-γ (IFN-γ), TNFα and lymphotoxin (LTα, LTβ and LIGHT) signaling pathways are all very similar in CTLs under the two stimulation conditions (Fig. 2B). Taken together, our data suggest that the decreased tumor rejection efficacy of CD3 mAb-stimulated CTLs is not due to defects in activation of the anti-tumor cytotoxic effecter mechanisms.

### Cognate Ag stimulation is required to maintain the CTL proliferative potential

**Figure 3:**
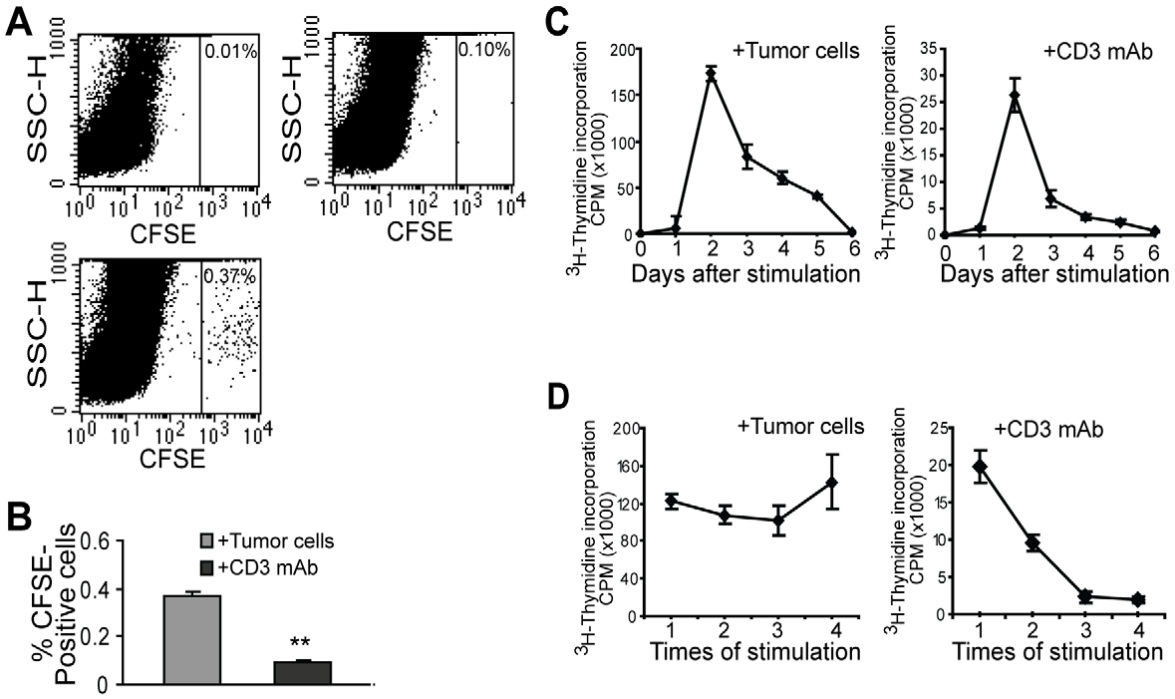
Ag stimulation maintains CTL persistence and proliferative potential. **A.** CTL persistence *in vivo* after adoptive transfer. Tumor cell- or CD3 mAb-stimulated CTLs were labeled with CFSE and injected into tumor-bearing mice. Four days later, lungs were excised from control mice (no CTL transfer, top left panel), and mice that received CD3 mAb- (top right panel) or tumor cell-stimulated- (bottom panel) CTLs, respectively. Lungs were digested into single cell suspension and analyzed for CFSE-positive cells by flow cytomotery. **B**. The percent of CFSE-positive cells as measured in A were quantified. *Column*, mean; *bar*, SD. ** *p*<0.001. n = 5 mice. **C.** Kinetics of CTL proliferation after activation. CTLs were stimulated with tumor cells or CD3 mAb and analyzed for ^3^H-thymidine incorporation over a 6-day period. **D**. Persistence of proliferation potential of CTLs after repeated stimulation. CTLs were stimulated with irradiated tumor cells or CD3 mAb every 7 days for a total of 4 rounds. ^3^H-thymidine incorporation was measured at day 2 after each re-stimulation.

Our above observations suggest that the poor efficacy of the CD3 mAb-activated CTLs to reject tumor is not due to the activation status of the CTLs. This raises the possibility that this inability might be due to lack of persistence of the CTLs after transfer. To test this possibility, we labeled tumor- and CD3 mAb-activated CTLs with CFSE tracking dye prior to transfer into tumor-bearing mice. Four days after CTL transfer, we excised tumor-bearing lungs and made single cell suspensions. The survival of adoptively transferred CTLs was examined by analysis of the CFSE-positive CTLs in the lung tumor. The rationale is that if CTLs survive in the tumor microenvironment, they can be detected as CFSE-positive cells. Analysis of cell suspensions from the tumor-bearing lungs revealed that indeed the tumor cell-activated CTLs are present in the tumor-bearing lungs (Fig. 3A&B), in contrast, much less CD3 mAb-stimulated CTLs remain in the tumor-bearing lungs.

The lack of persistence of the CD3 mAb-activated CTLs in the tumor-bearing hosts suggests that these CTLs either failed to proliferate or simply died *in vivo* [38], [39]. To determine whether cognate Ag or CD3 mAb induce different proliferation profiles of the tumor-specific CTLs, we examined the proliferation kinetics of the tumor-specific CTLs after activation. The tumor cell-stimulated and CD3 mAb-activated CTLs exhibited almost identical proliferation kinetics, however, the proliferation rate is greater in the tumor cell-stimulated CTLs than the CD3 mAb-stimulated CTLs (Fig. 3C). More importantly, CTLs exhibited sustained proliferation after repeated Ag stimulation, whereas repeated stimulation with CD3 mAb resulted in the quick decline of the proliferation potential of the CTLs (Fig. 3D).

**Figure 4:**
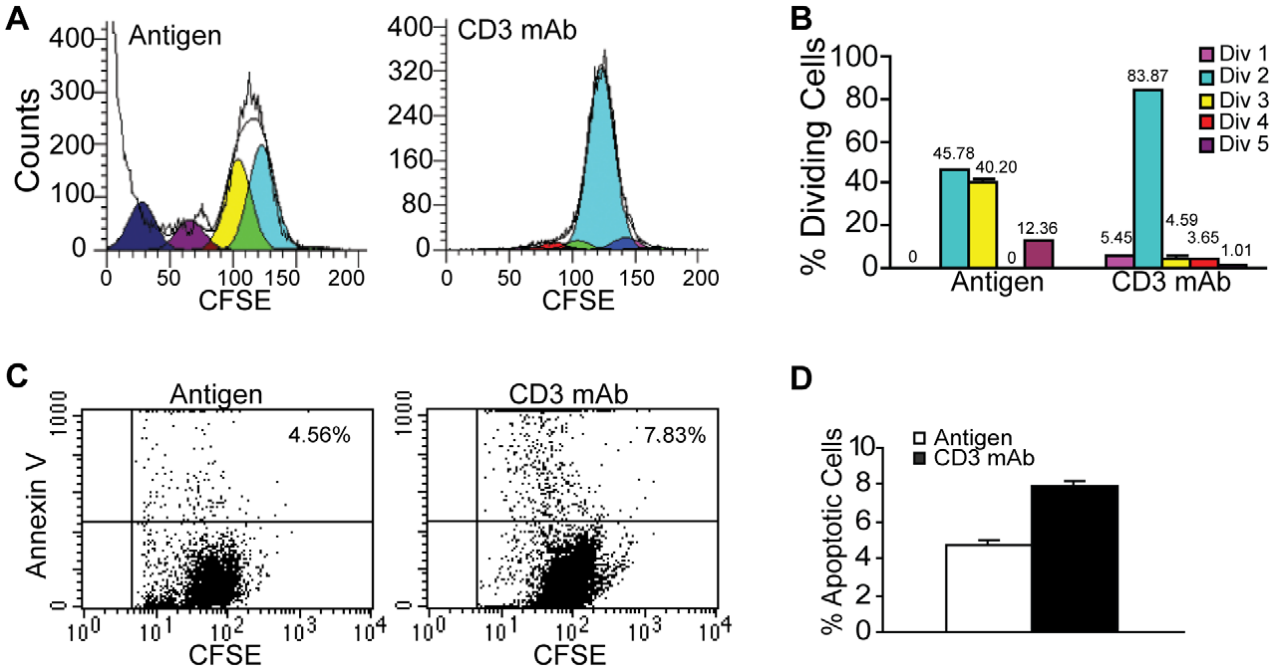
Antigen and CD3 mAb induce different proliferation and apoptosis profiles of tumor-specific CTLs. **A–B.** Proliferation profiles of tumor-specific CTLs. CTLs were labeled with CFSE and stimulated with either antigen or CD3 mAb. CTLs were analyzed for CFSE intensity 3 days after stimulation. Representative proliferation profiles are shown in A. Each division population was then quantified and presented in B. The percentage of cells in each division population is indicated at the top of the bars. **C–D**. Analysis of apoptosis of antigen- and CD3 mAb-stimulated CTLs. The stimulated CTLs as described in A–B were stained with Annexin V and analyzed by flow cytometry. Representative apoptosis profiles are shown in C. The percentage of apoptotic cells as shown in C were quantified and presented in D. *Column*, mean; *bar*, SD.

Activation of T cells often results in activation-induced cell death [37]. To determine whether antigen- and CD3 mAb-stimulated cells exhibit different apoptosis rates, the CFSE-labeled CTLs were stimulated with either the AH1 peptide or CD3 mAb and analyzed for cell division and apoptosis. AH1 peptide is the peptide that contains the epitope recognized by the CTLs used in this study [23]. Use of peptide instead of tumor cells reduces the background for apoptosis analysis. Consistent with what was observed in ^3^H incorporation analysis, antigen stimulation induced faster proliferation rates than CD3 mAb stimulation (Fig. 4A). However, antigen-stimulated CTLs exhibited less apoptosis than CD3 mAb-stimulated CTLs (Fig. 4B). Thus, antigen stimulation leads to greater proliferation and less apoptosis of the tumor-specific CTLs *in vitro.*

**Figure 5:**
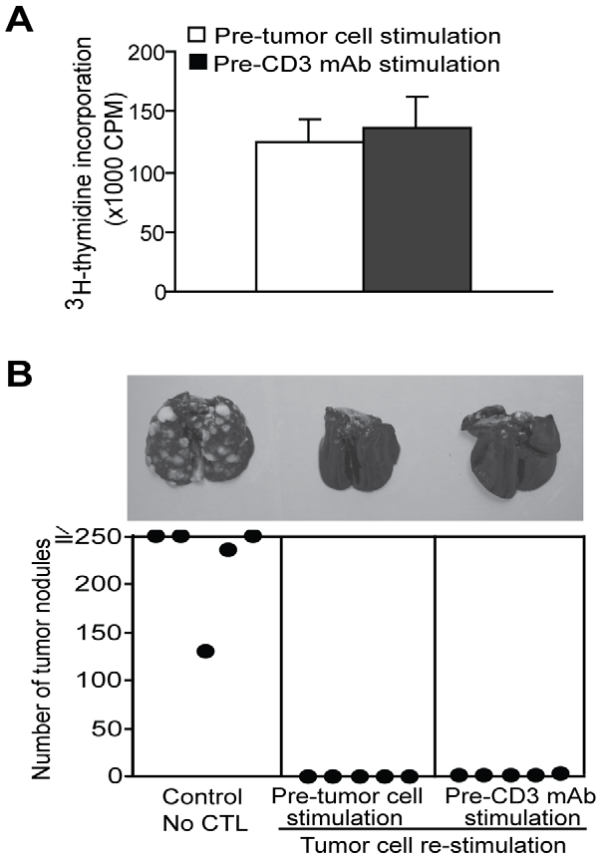
Loss of proliferative potential and anti-tumor cytotoxicity in CTLs is reversible. **A**. The loss of proliferation potential of CD3 mAb-stimulated CTLs is reversible. The tumor-specific CTLs were re-stimulated with CD3 mAb for 2 times, followed by re-stimulation with tumor cells. Proliferation was measured by analyzing ^3^H-thymidine incorporation 2 days after the second tumor cell re-stimulation. *Column*, mean; *bar*, SD. **B**. The decreased anti-tumor cytotoxicity of CD3 mAb-stimulated CTLs can be enhanced by antigen re-stimulation. CTLs were stimulated with tumor cells (Pre-tumor cell) or CD3 mAb (pre-CD3 mAb) every 7 days for 2 rounds, followed by re-stimulation with tumor cells twice more rounds before adoptive transfer. Shown are photomicrographs of individual lungs of mice. The number of tumor nodules was quantified and shown in the bottom panel. Each dot represents total tumor nodule counts from a single mouse.

To determine whether the reduced proliferation potential of the CD3 mAb-stimulated CTLs is reversible, we stimulated the CTLs with CD3 mAb for two rounds, and then re-stimulated the CTLs with tumor cells. CTLs were then analyzed for proliferation *in vitro* and tumor rejection efficacy *in vivo*. It is clear that re-stimulation of CD3 mAb-stimulated CTLs with antigen can restore the proliferation potential of the CTLs (Fig. 5A). Consistent with the restored proliferation potential, CD3 mAb-stimulated cells exhibited potent anti-tumor cytotoxicity *in vivo* after re-stimulation with the cognate Ag (Fig. 5B). Thus, we conclude that CD3 mAb stimulation does not result in senescence and the reduced proliferation potential of the CD3 mAb-stimulated CTLs is reversible.

### *Ifi202* and *survivin* are differentially induced by cognate Ag or CD3 mAb

**Figure 6:**
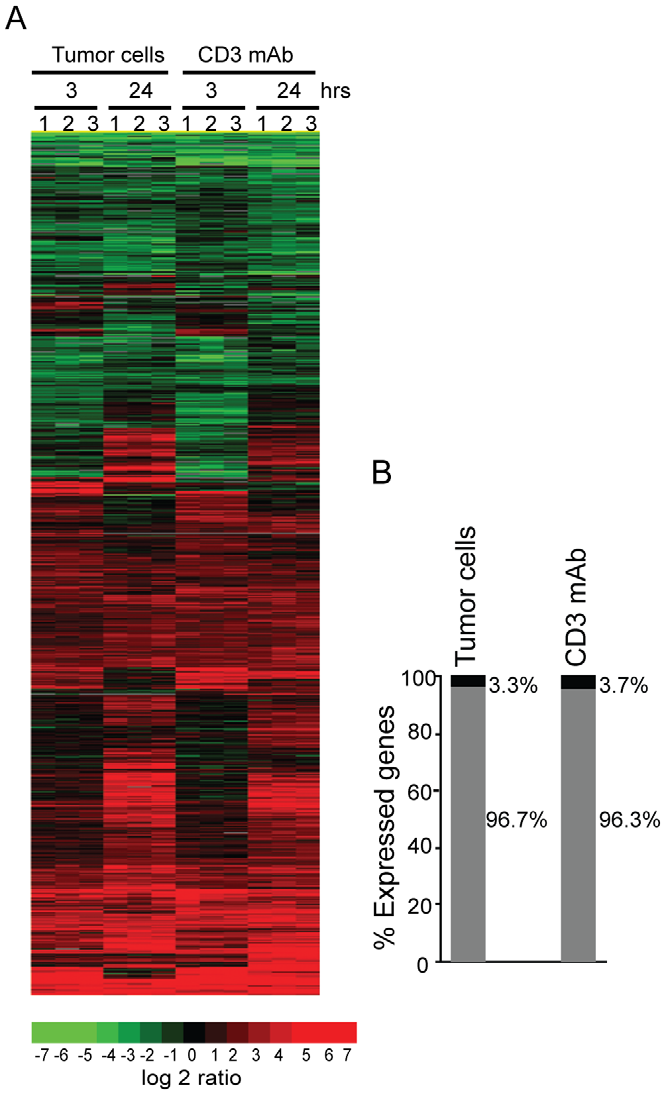
Gene expression profiles of the tumor-specific CTLs after tumor cells and CD3 mAb stimulation. **A.** CTLs were either stimulated with tumor cells or with CD3 mAb for the indicated time and analyzed for the genome-scale gene expression profiles using DNA microarray. Genes whose expression levels are changed by at least 2 fold (either up-regulated or down-regulated) at either time point or with either stimulation were selected. Cluster 3.0 program was used to analyze the gene expression patterns in a one-dimensional hierarchical clustering to generate gene dendrograms based on the pair-wise calculation of the Pearson coefficient of normalized fluorescence ratios as measurements of similarity and linkage clustering. The clustered data were loaded into TreeView program and displayed by the graded color scheme. **B.** Differential gene expression profiles between tumor cell-stimulated and CD3 mAb-activated CTLs. Genes whose expression levels were either up-regulated or down-regulated more than 2 fold in tumor cell-activated CTLs but not in CD3 mAb-activated CTLs were selected as tumor cell-regulated unique genes. Genes whose expression levels were either up-regulated or down-regulated more than 2 fold in CD3 mAb-activated CTLs, but not in tumor cell-stimulated CTLs, were selected as CD3 mAb-regulated unique genes. By these selection criteria, 93% genes are expressed at similar levels between tumor cell- or CD3 mAb-activated CTLs. Approximately 3.3% (a total of 392 genes/transcripts) are antigen-regulated unique genes and 3.7% (a total of 438 genes/transcripts) are CD3 mAb-regulated unique genes.

Our data suggest that cognate Ag or CD3 mAb stimulation results in different proliferation potentials and susceptibility to apoptosis of the tumor-specific CTLs (Fig. 3&4). To gain a better understanding of the molecular basis of the different proliferative potentials of CTLs, we conducted genome-wide gene expression profiling of the CTLs after stimulation with tumor cells or CD3 mAb, respectively. DNA microarray analysis revealed that, although their anti-tumor efficacy is dramatically different *in vivo*, the gene expression kinetics of the tumor-specific CTLs stimulated with tumor cells or CD3 mAbs are surprisingly very similar (Fig. 6). The expression levels of a total of 11964 (out of 36212 genes on the array chip) were identified to be altered at least 2 fold at either 3 or 24 h after stimulation with tumor cells or CD3 mAb. However, only 392 genes (3.3%) of the 11964 genes were uniquely regulated by cognate Ag stimulation, and only 438 genes (3.7%) of the 11964 genes were differentially regulated by CD3 mAb stimulation (Fig. 6B). Taken together, our data suggest that cognate Ag and CD3 mAb induce overall similar gene expression profiles in the tumor-specific CTLs. Nevertheless, a small group of genes are uniquely associated with cognate Ag or CD3 mAb-induced activation.

**Figure 7:**
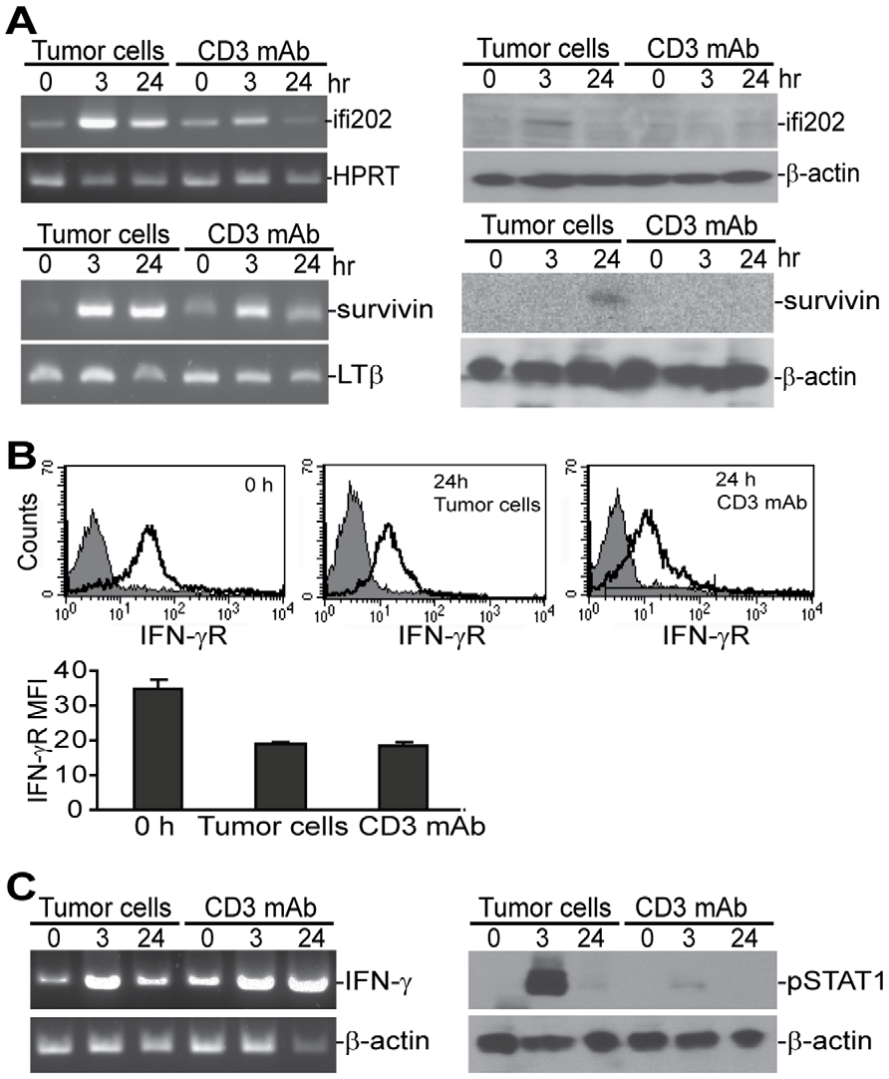
*Ifi202* and *survivin* are differentially expressed between the tumor cell-and CD3 mAb-activated CTLs. **A**. RT-PCR and Western blotting analysis of *ifi202* and *survivin* expression level during CTLs activation. CTLs were stimulated with either tumor cells or CD3 mAb for the indicated time and analyzed by RT-PCR (left panel) and Western blotting (right panel). **B**. Cell surface IFN-γR staining of CTLs. CTLs were either un-stimulated (0 h) or stimulated with tumor cells or CD3 mAb for 24 h, respectively. The CTLs were then double stained with anti-CD8- and anti-IFN-γR-specific mAbs. The figure is representative of three IFN-γR staining profiles of CD8^+^ cells. Bottom panel: the mean fluorescent intensity (MFI) of IFN-γR was quantified and shown as mean±SD. *Column*: mean; *bar*, SD. *P*<0.001. **C**. STAT1 is preferentially activated in tumor cell-activated CTLs. CTLs were activated as in A and analyzed for IFN-γ transcript level by RT-PCR (left panel) and for phosphorylated STAT1 level by Western blotting analysis (right panel). The Western blot shown is representative of two separate experiments.

We next focused on genes with known functions in cellular proliferation and/or apoptosis. Two such genes: *ifi202* and *survivin*, were identified. Western blotting and RT-PCR analysis confirmed that the degree of up-regulation of both *ifi202* and *survivin* are greater in tumor-activated CTLs than in CD3 mAb-activated CTLs (Fig. 7A). Because expression of *ifi202* is predicted to be up-regulated by IFN-γ [40], [41], [42], we next sought to determine whether differential *ifi202* activation is due to different IFN-γR levels in the CTLs after tumor cells or CD3 mAb stimulation. Flow cytometry analysis indicates that IFN-γR is highly expressed in the CTLs and stimulation resulted in decreased IFN-γR level in the CTLs. However, tumor cell- and CD3 mAb-stimulated CTLs expressed similar levels of IFN-γR (Fig. 7B). IFN-γ was activated in the CTLs after stimulation and the IFN-γ activation kinetics are similar under the two activation conditions (Fig. 7C). Thus, the observed difference in expression levels of *ifi202* between tumor cell- and CD3 mAb-stimulated CTLs are unlikely due to a difference in IFN-γR levels expressed by the CTLs. IFN-γ binding to the IFN-γR leads to STAT1 phosphorylation. The phosphorylated STAT1 dimerizes and translocates to the nucleus to activate target genes [43]. To determine whether STAT1 is differentially activated in CTLs, we examined STAT1 phosphorylation kinetics in the CTLs after activation. Western blotting analysis revealed that STAT1 is quickly activated in tumor cell-stimulated CTLs, but not in CD3 mAb-stimulated CTLs (Fig. 7C). Taken together, our data suggest that the cognate Ag stimulation induces STAT1 activation, whereas CD3 mAb is unable to activate STAT1 and thus results in defective IFN-γ signaling in the CTLs, which might be responsible, at least in part, for the lack of *ifi202* and *survivin* up-regulation in the activated CTLs.

### STAT1 directly regulates *survivin* and *ifi202* expression

**Figure 8:**
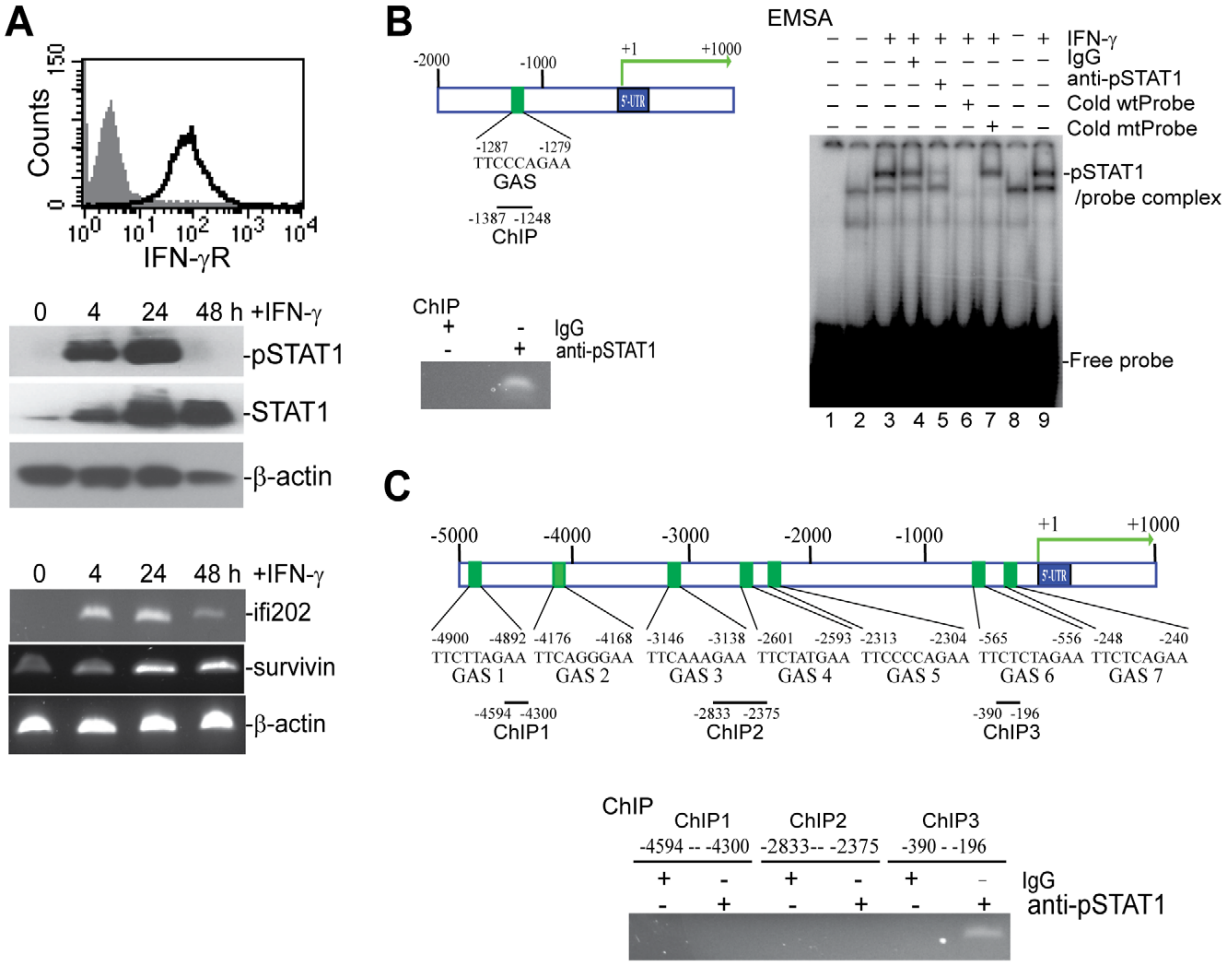
*Ifi202* and *survivin* expression is directly regulated by the IFN-γ signaling pathway. **A**. IFN-γ induces *ifi202* and *survivin* expression in T cells. T cell line 5KC was stained for IFN-γR protein level (top panel), and treated with IFN-γ for the time indicated and analyzed for STAT1 activation (middle panel) and induction of *ifi202* and *survivin* (bottom panel). **B**. IFN-γ-activated STAT1 (pSTAT1) binds to the *survivin* promoter region. Top left panel: *survivin* promoter structure. Number above the bar indicates nucleotide positions relative to *survivin* transcription initiation site. The GAS element location and the ChIP PCR region are indicated under the bar. Bottom left panel: ChIP analysis of pSTAT1 association with the *survivin* promoter. 5KC cells were treated with IFN-γ for approximately 24 h and analyzed by ChIP with pSTAT1-specific mAb and *survivin* promoter-specific PCR. Right panel: EMSA of pSTAT1 and the GAS element of the *survivin* promoter. Nuclear extracts were prepared from untreated (lanes 2 and 8) and IFN-γ-treated (lanes 3–7 and 9) 5KC cells. Nuclear extracts were incubated in the presence of isotype control IgG mAb (lane 4), anti-pSTAT1 mAb (lane 5), excess cold probe (lane 6) and mutant cold probe (lane 7). Lanes 1–7: probe of *survivin* promoter GAS element. Lanes 8 and 9: probe of IRF8 GAS element. **C**. IFN-γ-activated STAT1 binds to the *ifi202* 5′-regulatory region. Top panel: *ifi202* regulatory region structure. Number above the bar indicates nucleotide positions relative to *ifi202* transcription initiation site. The GAS elements and the ChIP PCR-amplified regions are indicated under the bar. Bottom panel: ChIP analysis of pSTAT1 association with the *survivin* promoter. 5KC cells were treated with IFN-γ for approximately 24 h and analyzed by ChIP with pSTAT1-specific mAb and *ifi202* gene-specific PCR.

Our above observations indicate that *survivin* expression in the tumor-specific CTLs is not only differentially induced by tumor cells and CD3 mAb stimulation (Fig. 7A) but also correlated with STAT1 activation (Fig. 7C), suggesting that *survivin* might also be a target gene of the IFN-γ signaling pathway. To test this hypothesis, we used the T cell line 5KC [24] to examine the role of the IFN-γ signaling pathway on *survivin* expression. Flow cytometry analysis indicates that IFN-γR is highly expressed on the cell surface of 5KC cells, and treatment of 5KC cells with recombinant IFN-γ induced STAT1 phosphorylation (pSTAT1) and increased STAT1 expression (Fig. 8A). Furthermore, IFN-γ treatment also increased *ifi202* and *survivin* expression (Fig. 8A). Thus, 5KC T cells resemble the tumor-specific CTLs in terms of IFN-γ signaling and *survivin* and *ifi202* expression, and thus is a model system to study regulation of *ifi202* and *survivin* expression. pSTAT1 is a transcription factor that binds to a consensus sequence termed Gamma Activation Sequence or GAS (TTC(n2-4)GAA) [44]. To determine whether pSTAT1 binds directly to the *survivin* gene promoter, we identified the *survivin* gene sequence from the mouse genome sequence database. Analysis of the promoter region of the *survivin* gene using the MacVector program identified a GAS element located between -1287 to -1279 relative to the *survivin* gene transcription initiation site (Fig. 8B). Analysis of pSTAT1 association with the chromatin region containing this GAS element using ChIP revealed that pSTAT1 is associated with this region of the chromatin in 5KC cells (Fig. 8B). To determine whether pSTAT1 binds directly to this GAS DNA element of the *survivin* promoter, we synthesized oligonucleotides containing this GAS element and made a double-stranded probe. Incubation of nuclear extract derived from IFN-γ-treated 5KC cells and analysis of pSTAT1 and the GAS probe interaction using EMSA showed that pSTAT1 binds specifically and directly to this GAS element (Fig. 8B). Taken together, our data suggest that *survivin* is a direct target gene of the IFN-γ signaling pathway during T cell activation.

Although expression of the *ifi202* is predicted to be up-regulated by IFN-γ [42], it is not fully understood how IFN-γ regulates *ifi202* expression. We analyzed the 5′-regulatory region of the *ifi202* gene and identified 7 potential GAS elements in the upstream 5000 nucleotide sequence relative to the potential *ifi202* gene transcription initiation site (Fig. 8C). ChIP analysis indicated that pSTAT1 is associated with the promoter region near the transcription start site (Fig. 8C). Thus, IFN-γ-activated STAT1 is also directly associated with the *ifi202* 5′-regulatory region to regulate *ifi202* expression.

### Silencing *ifi202* expression decreased T cell proliferation

**Figure 9:**
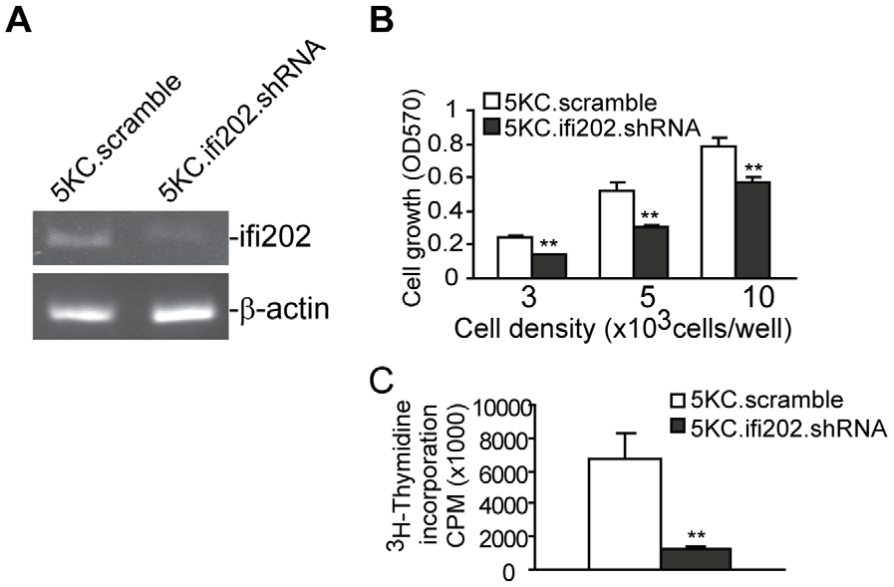
Silencing *ifi202* expression decreased T cell proliferation. **A**. Silencing of *ifi202* expression by *ifi202*-specific shRNA. 5KC cells were transduced with scramble shRNA-expressing lentiviral particles (5KC.scramble) and *ifi202*-specific shRNA (5KC.ifi202.shRNA), respectively. The transduced cells were selected with puromycin for stable lines and analyzed for *ifi202* expression by RT-PCR. **B**. Silencing *ifi202* expression decreased T cell proliferation. 5KC.scramble and 5KC.*ifi202*.shRNA cells were seeded in 96-well plates at the indicated density in the presence of IFN-γ (100 U/ml) for approximately 48 h and analyzed by MTT assay. ** *p*<0.01. **C**. Cells were seeded in a 96-well plate as described in B at a density of 3×10^3^ cells/well and measured for ^3^H incorporation. ** *p*<0.01.

It is known that increased expression of *ifi202* inhibits apoptosis [41], [45], [46]. We observed here a positive correlation between p202 protein level and CTL persistence (Fig. 7). To determine whether the p202 protein mediates T cell proliferation and survival, we silenced the *ifi202* expression with *ifi202*-specific shRNA (Fig. 9A). It is clear that silencing *ifi202* expression significantly inhibited 5KC T cell proliferation (*p*<0.01). Thus, *ifi202* plays a direct role in T cell proliferation and/or survival.

## Discussion

It is a well-established concept that T cells require Ag and co-stimulation signals for activation and proliferation. It has also been shown that a third molecular signal such as IL-2 is required for optimal T cell activation and proliferation [20]. In this study, we observed that stimulation of tumor-specific CTLs through the TCR/CD3 complex and co-stimulation molecule CD28 resulted in suboptimal proliferation and subsequent loss of proliferative potential. This activation-induced non-responsiveness is apparently not due to lack of IL-2 production by the activated CTLs since recombinant IL-2 is included in the culture medium. Furthermore, this non-responsive state is reversible by Ag stimulation, suggesting that the non-responsive CTLs are not senescent. It is known that re-stimulation of T lymphocytes through the TCR/CD3 complex also induces activation-induced cell death (AICD) and AICD is mediated by Fas/FasL-mediated apoptosis [47]. In this study, we observed that FasL is quickly up-regulated in both Ag- and CD3 mAb-stimulated CTLs (Fig. 2B) and a portion of the proliferating CTLs are apoptotic (Fig. 4C). It seems that CD3 mAb stimulation results in less proliferation and more apoptosis than Ag stimulation, which might at least partially explain the decreased tumor rejection efficacy of the CD3 mAb-stimulated CTLs.

To elucidate the molecular mechanisms underlying CD3 mAb stimulation-induced non-responsiveness of the tumor-specific CTLs, we carried out genome-wide gene expression analysis and observed that *ifi202* and *survivin*, two genes with known function in apoptosis and proliferation, are differentially expressed between Ag- and CD3 mAb-stimulated CTLs. *Ifi202* is an IFN-inducible protein and plays a role in the regulation of apoptosis [41], [42], [45]. Inhibition of the endogenous *ifi202* increased susceptibility to apoptosis induction [48], and overexpression of *ifi202* in cells induced to undergo p53-dependent apoptosis significantly delayed this process [49], suggesting that *ifi202* functions as an apoptosis inhibitor. In this study, we observed that *ifi202* is quickly up-regulated in tumor-specific CTLs after antigenic stimulation. Therefore, the lack of *ifi202* up-regulation in CD3 mAb-stimulated CTLs might contribute to the lack of CTL persistence in the tumor microenvironment (Fig. 3A) and decreased tumor rejection efficacy *in vivo* (Fig. 1).

*Survivin* expression is strictly regulated in T cells and plays an essential role in T cell maturation, survival and proliferation [50], [51], [52], [53], [54], [55], [56]. *Survivin*-deficient cells exhibited cell cycle arrest and increased cell death [52], and blocking *survivin* suppressed T cell proliferation and led to apoptosis [53]. These observations thus suggest that *survivin* plays an important role in T cell maturation, activation and apoptosis. Consistent with these observations, Ag-reactive T cells expressing *survivin* exhibited long-term survival and displayed enhanced anti-tumor activity [55]. In this study, we observed that *survivin* is rapidly up-regulated in tumor-specific CTLs after antigenic stimulation. Up-regulation of *survivin* is associated with sustained proliferation potential of the CTLs and is correlated with tumor-specific CTL persistence in the tumor microenvironment and tumor rejection efficacy *in vivo*. CD3 mAb stimulation apparently activated the tumor-specific CTLs (Fig. 2A) and the cytotoxic effecter mechanisms (Fig. 2B). However, CD3 mAb-activated tumor-specific CTLs failed to up-regulate *survivin*, which might explain, at least partially, why CD3 mAb-activated tumor-specific CTLs failed to persist in cancer patients after adoptive transfer [14].

The above studies strongly suggest that the expression of *survivin* is tightly regulated during T cell activation. However, the molecular mechanisms underlying the regulation of *survivin* expression are not entirely clear. Our study indicates that *survivin* is directly regulated by IFN-γ. Engagement of the IFN-γR leads to receptor α-chain dimerization, association of β-chains, transphosphorylation of the receptor-associated JAK kinases, and ultimately to phosphorylation and dimerization of STAT1, which is then translocated to the nucleus as an active transcription factor [57]. Therefore, STAT1 is a key mediator of the IFN-γ-initiated signaling pathway. We demonstrated that IFN-γ-activated STAT1 binds directly to a GAS element in the *survivin* promoter region to activate *survivin* expression (Fig. 8). IFN-γ is secreted by activated T cells (Fig. 7C). Thus, *survivin* expression during T cell activation is regulated by the activated T cell-secreted IFN-γ in an autocrine manner. Our data indicate that IFN-γ can quickly induce STAT1 phosphorylation in antigen-activated CTLs but not in CD3 mAb-activated CTLs (Fig. 7C). Therefore, the lack of survivin up-regulation in CD3 mAb-activated CTLs is likely due to the lack of STAT1 activation. The molecular mechanism underlying the failure of CD3 mAb to induce STAT1 activation remains to be determined. Nevertheless, our results indicate a novel role of IFN-γ during T cell activation: regulating *survivin* expression to maintain CTL proliferation potential and suppress apoptosis.

It has been shown that CD8^+^ effector cells expressing *survivin* and *bcl-xL* exhibit greater proliferation rates and long-term survival advantages. The *survivin* and *bcl-xL*-expressing T cells also exhibit greater tumor rejection efficacy [55]. We also observed that *survivin* up-regulation is associated with increased proliferation and enhanced tumor rejection efficacy. Furthermore, our data suggest that survivin expression in T cells is regulated by IFN-γ. Therefore, survivin-mediated T cell proliferation potential might be a general phenomenon. The role of bcl-xL in regulating tumor-specific CTL proliferation and tumor rejection efficacy is not investigated in this study.

It has been well-demonstrated that IFN-γ plays an essential role in suppressing tumor development [1], [2], [58] and that IFN-γ signaling on tumor cells is important for IFN-γ-mediated anti-tumor activity. It is also known that IFN-γ/STAT1 signaling on host immune cells is essential for the development of anti-tumor lytic effecter cells [59]. Type I IFNs have also been shown to provide a third signal directly to CTLs via a STAT4-dependent pathway for CTL survival, function and IFN-γ production [60]. However, how IFN-γ regulates effecter cells is not well-defined. It is known that co-stimulation is required for T cell proliferation, survival and differentiation. Co-stimulation induces expression of *bcl-xL* and *survivin* and thus promotes cell survival [55], [61]. In this study, we observed that stimulation of tumor-specific CTLs with CD3 mAb in the presence of a costimulation signal from CD28 failed to maintain CTL proliferation *in vitro* and persistence *in vivo*. In contrast, stimulation with tumor cells maintained CTL proliferation potential and persistence. Furthermore, CTL-produced IFN-γ directly up-regulates survivin and *ifi202* expression in an autocrine manner. Therefore, it seems that IFN-γ might function as a “third signal” to mediate CTL proliferation and survival [20], [60]. Our data suggest that IFN-γ might execute its anti-tumor function, at least partially, through regulating *survivin* and *ifi202* expression to maintain T cell persistence in the tumor microenvironment. We demonstrated that IFN-γ activates STAT1 to directly regulate *survivin* and *ifi202* expression during Ag-mediated T cell activation. Up-regulation of *survivin* might be essential for CTL proliferation potential and persistence in the tumor microenvironment, whereas up-regulation of *ifi202* might prevent or delay apoptosis of activated T cells. Thus, our observations suggest a novel function of the IFN-γ signaling pathway in maintaining CTL persistence and might aid in designing survivin and ifi202-based strategies to maintain CTL persistence to enhance CTL function in cancer immunotherapy.
